# Enterovirus A71 capsid protein VP1 increases blood–brain barrier permeability and virus receptor vimentin on the brain endothelial cells

**DOI:** 10.1007/s13365-019-00800-8

**Published:** 2019-09-11

**Authors:** Wenjing Wang, Jiandong Sun, Nan Wang, Zhixiao Sun, Qiyun Ma, Jun Li, Mingshun Zhang, Juan Xu

**Affiliations:** 1 Department of Infectious Disease, The First Affiliated Hospital of Nanjing Medical University, Nanjing, 210029 China; 2 Department of Infectious Disease, Nanjing First Hospital, Nanjing Medical University, Nanjing, 210029 China; 3 Department of Respiratory Medicine, People’s Hospital of Gaochun, Nanjing, 211300 China; 4 Key Lab of Antibody Technique of Health Ministry, Nanjing Medical University, Nanjing, 210016 China; 5 Department of Immunology, Nanjing Medical University, Nanjing, 210016 China

**Keywords:** Enterovirus A71, VP1, Blood-brain barrier, Claduin-5, Vimentin

## Abstract

Enterovirus A71 (EV-A71) is the major cause of severe hand-foot-and-mouth diseases (HFMD), especially encephalitis and other nervous system diseases. EV-A71 capsid protein VP1 mediates virus attachment and is the important virulence factor in the EV-A71pathogenesis. In this study, we explored the roles of VP1 in the permeability of blood–brain barrier (BBB). Sera albumin, Evans blue, and dextran leaked into brain parenchyma of the 1-week-old C57BL/6J mice intracranially injected with VP1 recombinant protein. VP1 also increased the permeability of the brain endothelial cells monolayer, an in vitro BBB model. Tight junction protein claudin-5 was reduced in the brain tissues or brain endothelial cells treated with VP1. In contrast, VP1 increased the expression of virus receptor vimentin, which could be blocked with VP1 neutralization antibody. Vimentin expression in the VP1-treated brain endothelial cells was regulated by TGF-β/Smad-3 and NF-κB signal pathways. Moreover, vimentin over-expression was accompanied with compromised BBB. From these studies, we conclude that EV-A71 virus capsid protein VP1 disrupted BBB and increased virus receptor vimentin, which both may contribute to the virus entrance into brain and EV-A71 CNS infection.

## Introduction

Hand-foot-and-mouth disease (HFMD) caused by enterovirus A 71 (EV-A71) is usually self-limited (Xu et al. 2010). In some cases, however, the aseptic meningitis, brain stem encephalitis, and other nervous system diseases in the EV-A71 infected infants may be life-threatening (Gu et al. 2017). As a selective barrier, blood–brain barrier (BBB) protects central nervous system (CNS) from harmful pathogens in the blood. The access of EV-A71 into the CNS, except via retrograde axonal transport by nerves, most likely occurs through the BBB (Feng et al. 2016). As a neurotropic virus, the mechanisms by which EV-A71 crossing the BBB and disseminating into brain parenchyma remain largely enigmatic (Denizot et al. 2012).

Virus may cross the BBB via transcellular pathway or paracellular pathway. In the transcellular pathway, the virus attaches with and gains the entrance into the brain endothelial cells. In the paracellular pathway, the virus penetrates through the junctions between the neighboring endothelial cells. Tight junctions are critical in determining BBB permeability. Among of tight junction proteins, claudin-5 and ZO-1 are of great importance. Lacking of claudin-5 may increase size-selected paracellular permeability for small molecules (Nitta et al. 2003). As a junctional adaptor protein, interacting with claudin-5 and ZO-1 controls adherens junctions and plays essential roles in the barrier formation (Tornavaca et al. 2015). The absence of ZO-1 on the BBB was observed in the encephalitis caused by human immunodeficiency virus type 1 (HIV-1) (Dallasta et al. 1999).

Though EV-A71 virus capsid is formed by VP1, VP2, VP3, and inner VP4 (Plevka et al. 2013), VP1 is the major virulence factor for EV-A71 entrance into host cells (Tee et al. 2010). Receptors for VP1 including scavenger receptor class B member 2 (SCARB2) (Yamayoshi et al. 2009), P-selectin glycoprotein ligand1 (PSGL-1) (Nishimura et al. 2009), annexin II protein (Yang et al. 2011), heat shock protein-70 (HSP-70) (Xu et al. 2019), and vimentin (Du et al. 2014) have been extensively explored in EV-A71 infection. Among these diverse receptors, vimentin is an abundant intermediate filament protein primarily in endothelial cells (Franke et al. 1982), modulating cell adhesions (Dave and Bayless 2014) and lymphocyte transcellular migration (Nieminen et al. 2006). In this study, we demonstrated that VP1 may promote EV-A71 entrance via the increased BBB permeability and vimentin expression on BBB. Moreover, vimentin over-expression on brain endothelial cells was accompanied with increased BBB permeability.

## Materials and methods

### Animals and ethics statement

Wild-type female C57BL/6J mice (1 week old) were purchased from the Comparative Medical Center, Yangzhou University (Yangzhou, China). All mice were maintained under controlled conditions of a 12-h light/dark cycle at 23 ± 1.5 °C. Animal experiments were approved by the Institutional Animal Care and Use Committee of the Nanjing Medical University. All animal protocols were reviewed by and approved by the Animal Care and Use Committee (IACUC) of Nanjing Medical University (1708004).

### VP1 protein intracranial injection animal model

EV-A71 VP1 (GenBank: ABS82575.1) recombinant protein (< 0.1 EU/μg endotoxin) was custom ordered from Genscript (Nanjing, China). Twenty-four experimental mice were randomly divided into four groups: blank group (blank), sham operation group (sham), natural saline group (NS), and VP1 group (VP1). The blank control group was left untreated. The sham operation group was pricked with syringe needles. NS or VP1 group was injected intracranially with NS or NS containing VP1 (1 μg of VP1 protein diluted in 30 μl of NS). The video of intracranial injection can be found at JOVE (1994). Briefly, the mice were restrained and the syringe needle was inserted into brain 5 mm behind the eye, approximately 3 mm off the midline of the skull. To explore the BBB damage, the mice were intraperitoneally injected with fluorescein isothiocyanate–dextran (FTIC-dextran, FD4, Sigma) in PBS (5 mg/ml; 10 mg/kg) (Dittmar et al. 2008) or Evans blue dye (E2129, Sigma) in PBS (2%; 2 ml/kg) (Wang et al. 2018). The fluorescence intensity of FD4 in brain tissue homogenization was calculated at excitation wavelength of 485 nm and emission wavelength of 528 nm using a fluorescence plate reader (Biotek synergy). And the fluorescence intensity of Evans blue was calculated at excitation wavelength of 620 nm and emission wavelength of 680 nm using a fluorescence plate reader (Biotek synergy).

### Cells

The brain endothelial cells bEnd.3 from ATCC were cultured in the complete medium DMEM (Gibco, New York, USA) supplemented with 10% fetal bovine serum (Gibco, New York, USA) and antibiotics (penicillin and streptomycin) (Gibco, New York, USA) at 37 °C in a humidified atmosphere with 5% CO_2_.

### Antibodies and reagents

The anti-vimentin (ab92547), anti-claudin 5 (ab15106), anti-ZO1 (ab216880), anti-TGFβ (ab170874), and anti-albumin (ab135575) were purchased from Abcam (Cambridge, UK). The anti-GAPDH (#5174), anti-NF-κB p65 (#8242), anti-NF-κB p65 (phospho-Ser536) (#3033), anti-Smad3 (#9523), anti-Smad3 (phospho-Ser423/425) (#9520), anti-STAT3 (#4904), and anti-STAT3 (phospho-Tyr705) (#9145) were purchased from Cell Signaling Technology (Beverly, MA). Anti-PSGL-1 (#557787) was from BDPharmigen (Shanghai, China). Anti-SCARB2 (27102-1-AP), Anti-Annexin II protein (11256-1-AP), and anti-HSP-70 (10995-1-AP) were from Proteintech (Wuhan, China). EV-A71 VP1 neutralizing antibody (Cat.No.40013-H136) was purchased from Sino Biological (Beijing, China). The NF-κB inhibitor (BAY 11-7082, S2913) (Mosteiro et al. 2016) and Smad3 inhibitor (SIS3 HCl, S7959) (Zhu et al. 2017) were purchased from Selleck (Shanghai, China). Fluorescein isothiocyanate–dextran (FD4) and dimethyl sulfoxide (DMSO) (V900090) were purchased from Sigma-Aldrich (MO, USA). The mouse vimentin cDNA plasmid (MR207446) and control blank plasmid (PS100001) were purchased from OriGene Technologies (Beijing, China). The Lipofectamine 3000™ (#L3000-015) was purchased from Invitrogen (MA, USA).

### Western blotting

Total protein from the brain tissues or cells was extracted by lysis with RIPA buffer (89901, Thermo Fisher Scientific, MA, USA) containing halt protease inhibitor cocktail (78430, Thermo Scientific, MA,USA) and was sonicated on ice 5 times for 15 s each time. Protein concentrations were determined by a BCA assay. After a brief centrifugation, the proteins in supernatants solubilized in 5× SDS-PAGE and boiled at 100 °C for 5 min. After gel electrophoresis, the gel was transferred to polyvinylidene difluoride (PVDF) membranes (Millipore, Billerica, USA). After blocking for 1 h at room temperature with 5% skim milk, the PVDF membranes were incubated at 4 °C overnight with primary antibodies. The next day, the membranes were incubated with goat anti-rabbit HRP IgG (EarthOx Life Sciences, CA) and then incubated for 1 h at room temperature. After washing with TBST, the antibody–antigen complexes was detected with Immobilon Western Chemiluminescent HRP Substrate (WBKLS0500, Millipore, MA, USA) and visualized using the G:Box gel doc system (Syngene, UK).

### Immunohistochemical staining

The brain tissues were fixed with 4% paraformaldehyde and embedded in paraffin. Tissue sections were stained with H&E or incubated with primary antibodies against albumin (1:300) or vimentin (1:200) in the dark overnight followed by incubation with horseradish peroxidase-conjugated secondary antibody. Positive IHC staining was presented as brown staining and observed by a Zeiss Axio Examiner microscope.

### Confocal microscopy and immunofluorescence

The bEnd.3 cells were seeded in confocal dish in a total volume of 2 ml complete medium and were stimulated with 0.1 μg/ml VP1 for 24 h. Then, bEnd.3 cells were washed with PBS, fixed with 4% paraformaldehyde for 30 min, blocked with 5% goat serum for 1 h, and incubated with primary antibodies against vimentin overnight at 4 °C. After incubation of the primary antibodies, bEnd.3 cells were stained with Alexa Fluor® 555 goat-anti-rabbit antibody (1:500, Life technologies, USA) in the dark for 2 h. At last, 4′6-diamidino-2-phenylindole (DAPI, 1:2000) was added for 20 min. Images were captured by ZEISS LSM710 confocal fluorescence microscope.

### Transmission Electron microscopy

The bEnd.3 cells were seeded into upper chamber of Transwell™ plates in a total volume of 0.6 ml media, and then, 0.1 μg/ml VP1 was added into the upper chamber for 24 h. Then, the cells were washed in PBS and fixed in 1% osmium tetroxide in phosphate buffer, dehydrated in graded ethanol solutions, treated in propylene oxide, and embedded in epoxy–resin embedding media. Sixty-nanometer thin transverse random sections were collected on single copper slot grids coated with parlodion, stained with uranyl acetate and lead citrate, and observed with a FEI Tecnai G2 Spirit Bio TWIN transmission electron microscope.

### TEER measurements

Trans-endothelial electrical resistance (TEER) was measured to evaluate the integrity of the in vitro BBB models. The bEnd.3 cells were seeded on each insert (upper compartment) in transwell™ plate and stimulated with 0.1 μg/ml VP1. The bEnd.3 cell monolayer was measured TEER everyday by using a Millicell® ERS-2 Electrical Resistance System according to manufacturer’s protocol. The TEER values of coated but cell-free inserts were subtracted from the measured TEER values, and the difference was multiplied with the size of the insert (0.3 cm^2^ for each 24-well insert).

### Neutralizing antibody test

The bEnd.3 cells were seeded into 6-well plate in a total volume of 2 ml complete medium. Experimental groups were randomly divided into four groups: control group (ctrl), VP1 group (VP1), VP1 + IgG group (ctrl-Ab), and VP1 + neutralizing antibody group (Anti-VP1). One microgram per milliliter human IgG was added into bEnd.3 cells in the VP1 + IgG group (ctrl-Ab). And 1 μg/ml VP1 neutralization antibody as added into bEnd.3 cells in the VP1 + neutralizing antibody group (Anti-VP1). One hour later, 0.1 μg/ml VP1 was added into the VP1 group (VP1), VP1 + IgG group (ctrl-Ab), or VP1 + neutralizing antibody group (Anti-VP1) for 12 h. Vimentin expression was detected by Western blotting.

### Inhibitor experiment

Experimental groups were randomly divided into four groups: control group (ctrl), VP1 group (VP1), VP1 + BAY group (BAY), and VP1 + SIS3 (SIS3). The NF-κB inhibitor (BAY) solution in DMSO (5 μM) or Smad3 inhibitor (SIS3 HCl) solution in DMSO (0.5 μM) was added into each well respectively. After 1 h, 0.1 μg/ml VP1 was added into each group for 12 h, expect for the control group. Proteins were extracted for the following Western blotting.

### Quantitative real-time PCR

Total RNA was obtained from the cells or fresh brain tissues with TRIzol Reagent (life technologies). And RNA was reverse-transcribed into cDNA with a reverse-transcribed kit (Abm, Zhenjiang, China) according to the manufacturer’s instructions. The RNA expression was quantified using a StepOnePlus Real-Time PCR System (ABI, USA). The primer sequences used for real-time PCR were designed by referring to PrimerBank. The primer sequences used were as follows.

- Vimentin-Forward: 5′-CCAGAGGGACCAGATGATCCA-3′
- Vimentin-Reverse: 5′-GGTGGCGAGTGATGTCCTG-3′
- GAPDH-Forward: 5′-AGGTCGGTGTGAACGGATTTG-3′
- GAPDH-Reverse: 5′-TGTAGACCATGTAGTTGAGGTCA-3′

### Plasmid transfection assay and measurement of bEnd.3 cells permeability

Vimentin plasmid cDNA or blank vehicle were transfected into bEnd.3 cells with Lipofectamine 3000 (Invitrogen, USA) according to the manufacture’s protocol. Transwell™ 24-well plates, clear inserts with 0.4 μm pore size of polyester membrane, and 6.5 mm diameter (Corning, NY, USA) were seeded with the transfected bEnd.3 cells into each insert (upper compartment). After the endothelial cell monolayer formed, FD4 solution in phosphate-buffered saline (PBS, 1 mg/ml) was added into the upper chambers for 12 h. The fluorescence intensity of FD4 in lower chambers was calculated at excitation wavelength of 485 nm and emission wavelength of 528 nm using a fluorescence plate reader (Biotek synergy).

### Statistical analysis

Statistical analysis was performed using Prism 7.0 (GraphPad Software, San Diego, CA, USA). The data were expressed as the mean ± standard error of the mean (SEM). The results were analyzed by one-way analysis of variance for repeated measures followed by Dunnett’s post hoc test to determine differences among multiple comparisons.

## Results

### VP1 increased BBB permeability in vivo

**Fig. 1:**
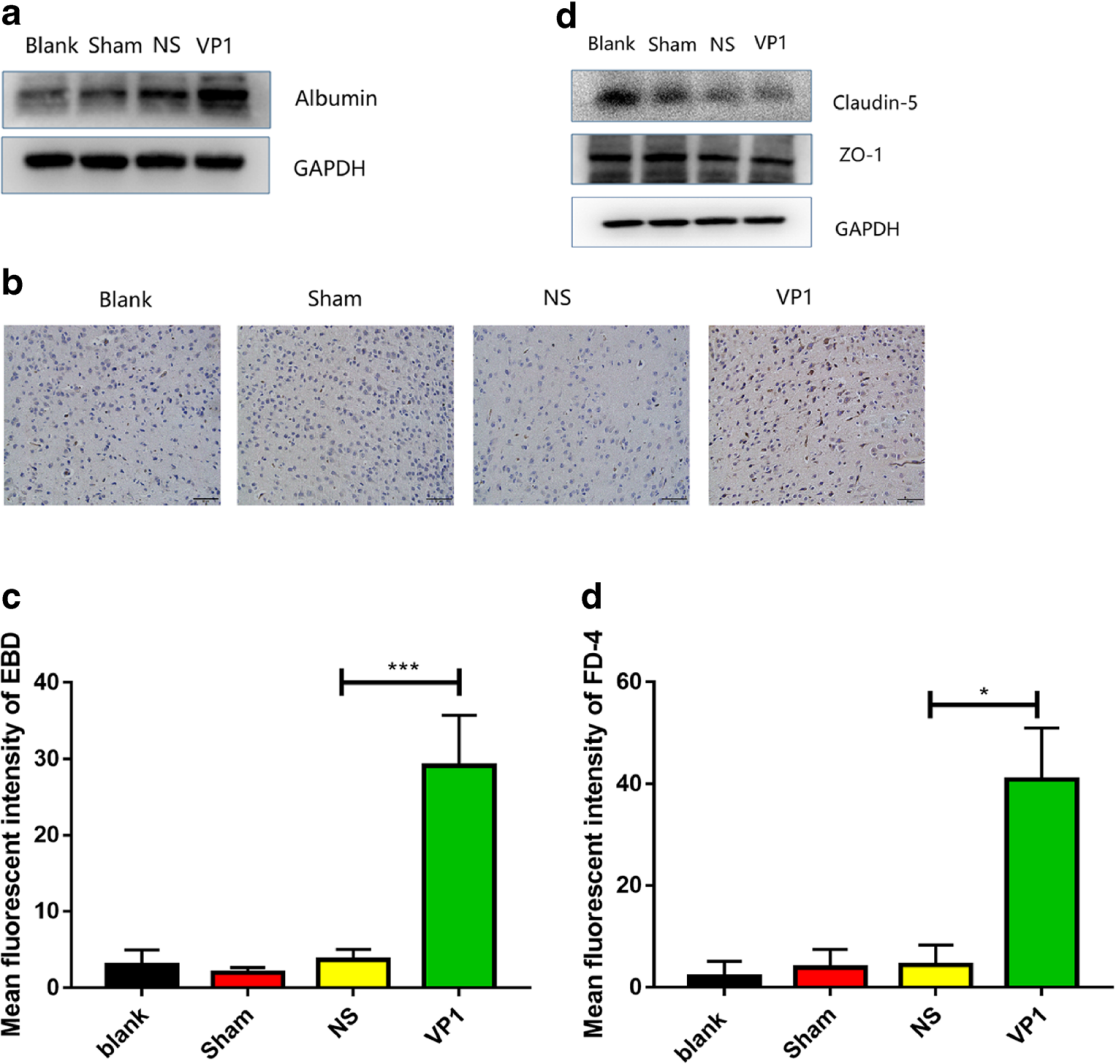
VP1 increased BBB permeability in vivo. **a** Western blotting showed the increased albumin in the brain tissues from the mice treated with VP1. **b** Immunohistochemistry staining revealed evident albumin in the brain tissues from the mice treated with VP1. **c**, **d** Fluorescence intensity of Evans blue dye (EBD) or FD4 was significantly elevated in the brain tissues from the mice treated with VP1. **e** Expression levels of tight junction proteins in the brain tissues. ZO-1 was comparable in the mice treated with normal saline or VP1. Claudin-5 was significantly reduced in the brain tissues treated with VP1. *p < 0.05

In the infants with brain stem encephalitis, VP1 expression was recorded in brain tissues (Li et al. 2015b). To explore the direct roles of VP1 in the EV-A71 pathogenesis, the recombinant VP1 protein was intracranially instilled into brain parenchyma. With the damage of BBB, albumin from blood may leak into brain tissues (Banks et al. 2000). Expectedly, VP1 caused the elevation of albumin in the brain parenchyma (Fig. 1a, b). Fluorescein isothiocyanate–dextran (FD4) and Evans blue in the brain parenchyma may also indicate the BBB impairment (Saunders et al. 2015). VP1 significantly promoted the FD4 and Evans blue leakage into brain parenchyma (Fig. 1c, d), implying that VP1 directly disrupt the integrity of BBB. Tight junction is essential in the maintenance of BBB integrity. In the brain parenchyma treated with VP1 or saline, the expression of ZO-1 was comparable. Claudin-5 expression, however, was markedly reduced in the brain tissues treated with VP1 (Fig. 1e).

### VP1 disrupted BBB in vitro

**Fig. 2:**
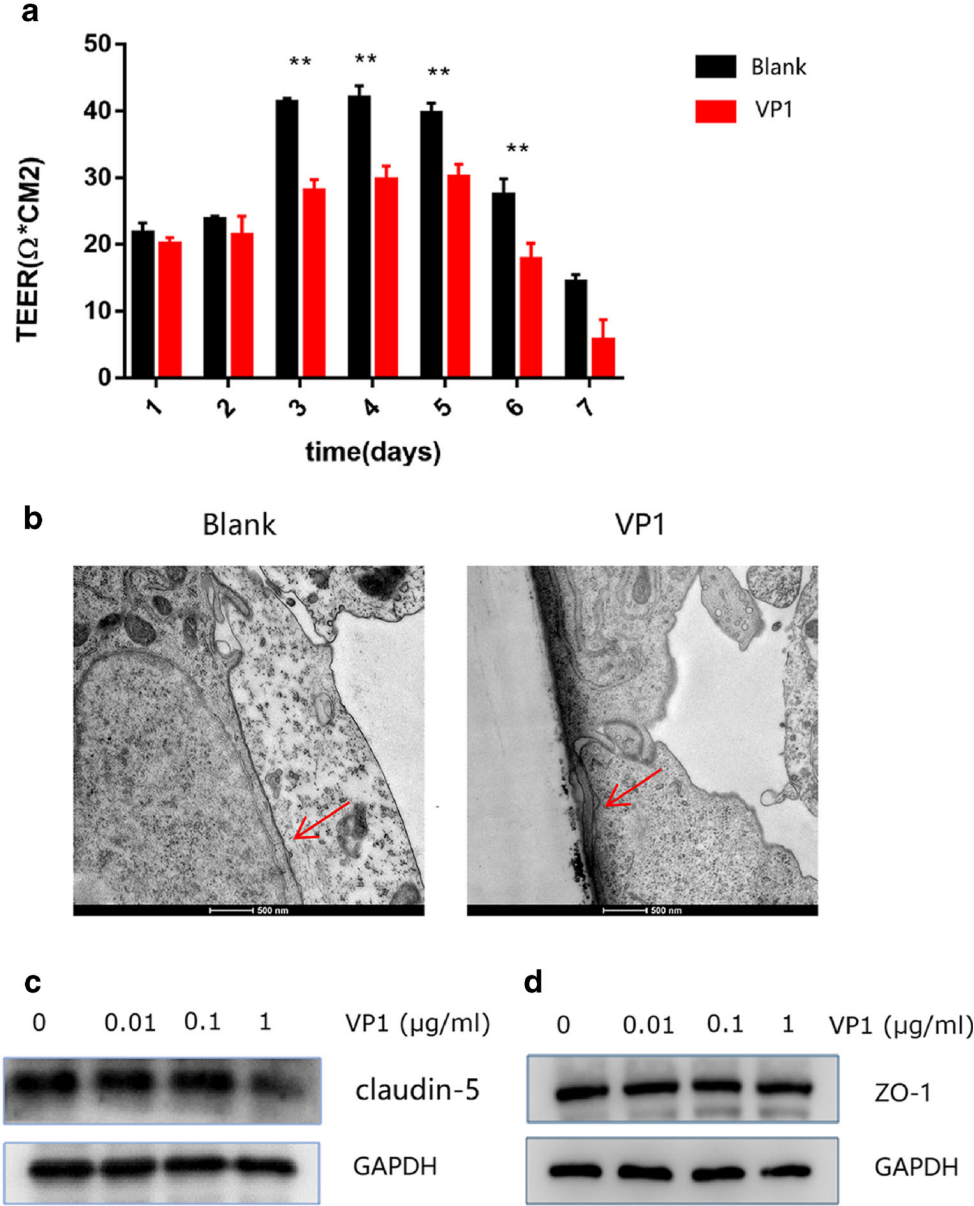
VP1 reduced claudin-5 and disrupted BBB in vitro. **a** VP1 significantly decreased the trans-endothelial electrical resistance (TEER). **b** VP1 damaged the tight junctions between endothelia cells observed with transmission electron microscope. **c**, **d** VP1 directly decreased claudin-5 in the brain endothelial cells; in contrast, ZO-1 was comparable in different groups. **p < 0.01

In vitro, with the brain endothelial cells growth and the formation of tight junctions, trans-endothelial electrical resistance (TEER) and the integrity of endothelial cell monolayer are gradually increased. Compared with normal saline, VP1 significantly decreased TEER, suggesting that VP1 damaged the BBB integrity (Fig. 2a). Under the transmission electron microscope, VP1 damaged the tight junctions between endothelia cells (Fig. 2b). Similar with in vivo observation, VP1 directly decreased claudin-5 in the brain endothelial cells in vitro (Fig. 2c), implying that claudin-5 may be the key target in the VP1-induced BBB leakage. ZO-1 expression in the different groups was similar (Fig. 2d). In sum, VP1 caused the reduction of tight junction protein claudin-5 and disruption of BBB.

### VP1 promoted the expression of vimentin on brain endothelia cells

**Fig. 3:**
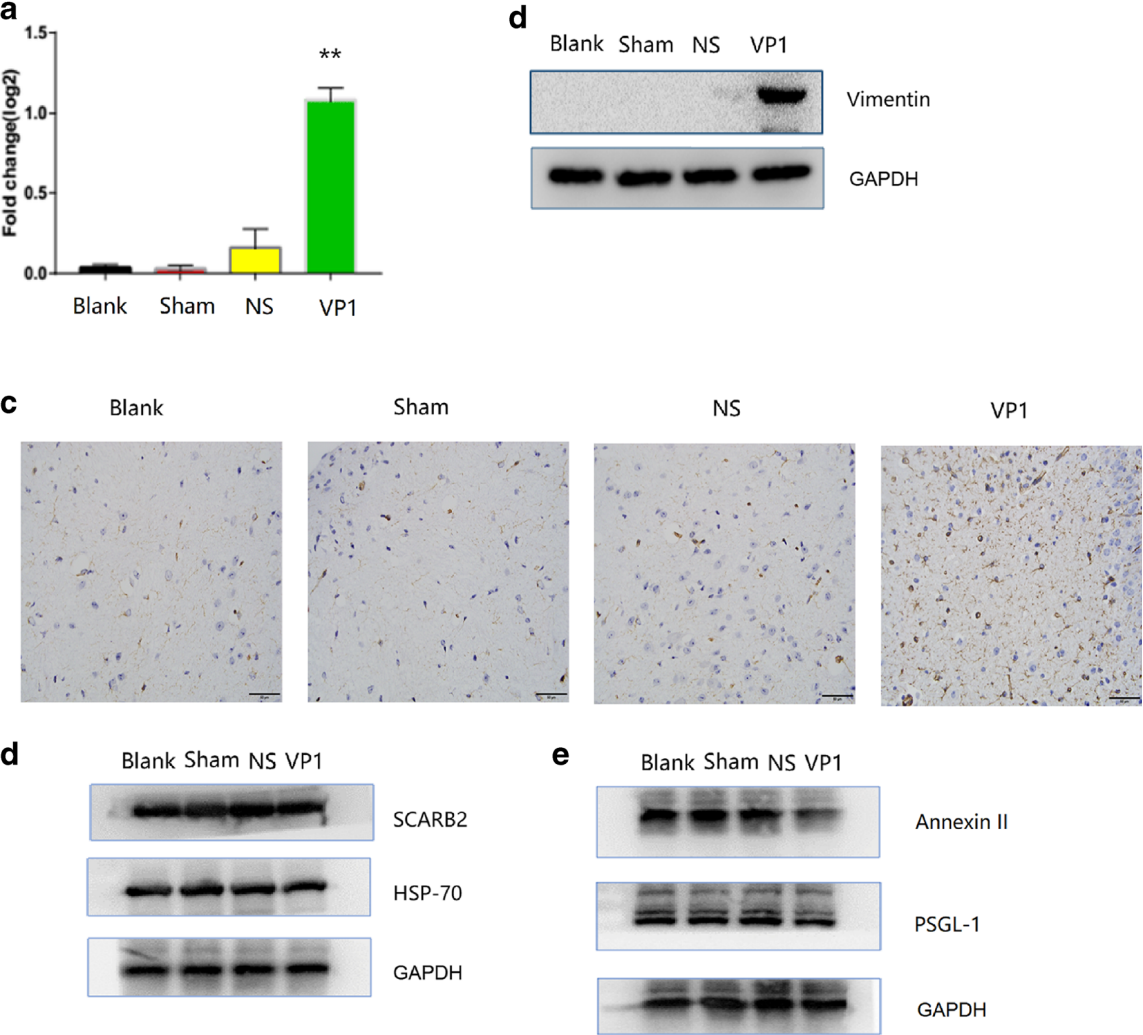
VP1 promoted the expression of vimentin in the brain tissues. **a** qRT-PCR showed the vimentin mRNA was significantly increased in the brain tissues from the mice treated with VP1. **b** Western blotting further showed the vimentin protein was significantly increased in the brain tissues from the mice treated with VP1. **c** In the immunohistochemistry analysis, vimentin was widely distributed in brain tissues and significantly increased upon VP1 challenge. **p < 0.01. **d** EV-A71 receptors SCARB2 and HSP-70 were almost unchanged on the brain endothelia cells treated with VP1. **e** EV-A71 receptor Annexin II was slightly decreased on the brain endothelia cells treated with VP1. Expression of PSGL-1 was comparable in the different groups

The increased permeability of BBB suggested that EV-A71 may gain the entrance into brain via the transcellular pathway. Besides the transcellular route, EV-A71 may bind with virus receptors and infect the endothelial cell. In the brain tissues treated with VP1, EV-A71 receptor vimentin was significantly increased (Fig. 3a, b). In the immunohistochemistry analysis, vimentin was widely distributed in brain tissues and significantly increased upon VP1 challenge (Fig. 3c). In contrast, EV-A71 receptors SCARB2, HSP-70, and PSGL-1 on the brain tissues treated with VP1 were almost unchanged, and Annexin II was even decreased (Fig. 3d, e), suggesting that vimentin may contribute to the VP1-meidated aggravation of EV-A71 CNS infection.

**Fig. 4:**
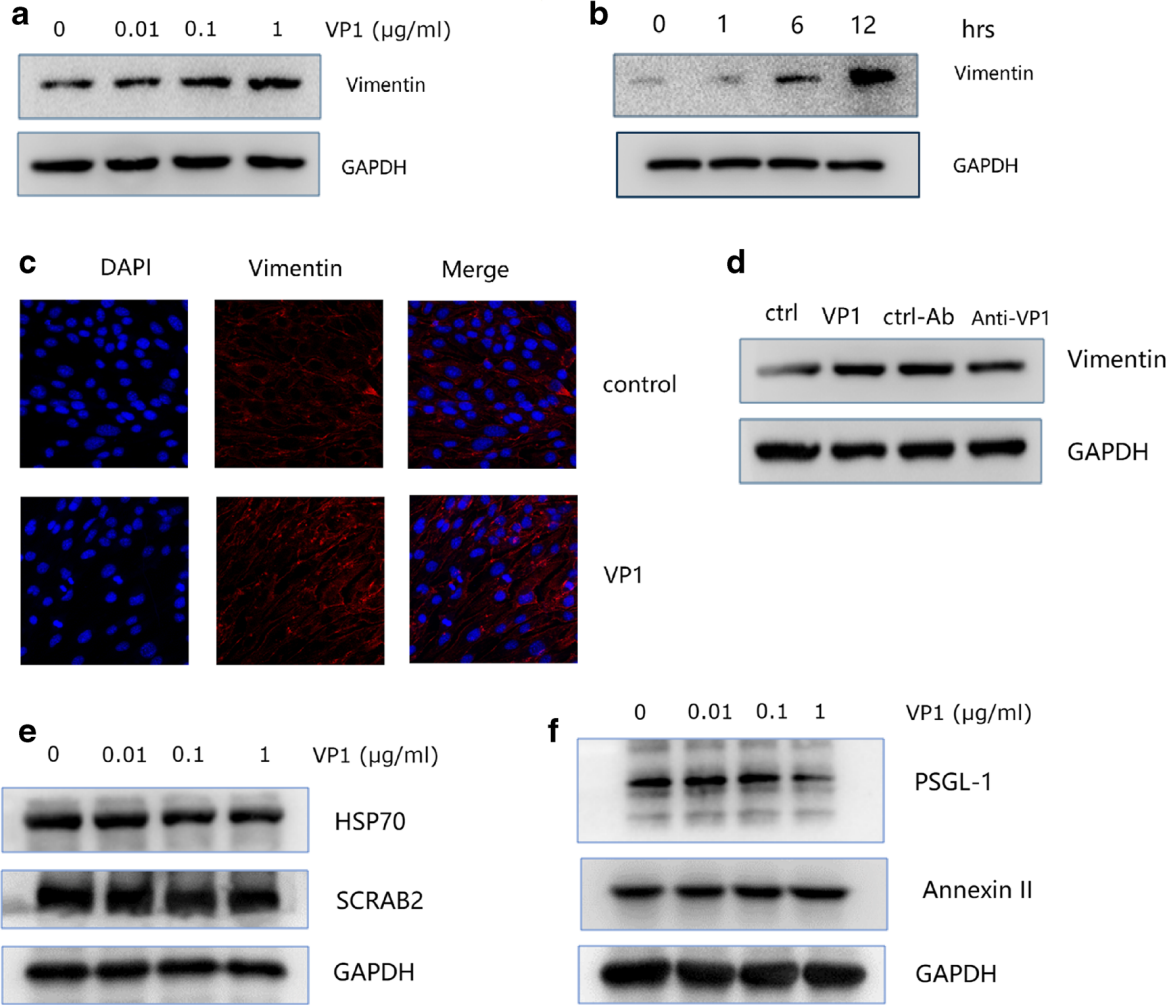
VP1 increased the expression of vimentin on the brain endothelial cells. **a** A dose-dependent increase of vimentin on the endothelial cells treated with VP1 for 24 h. VP1 with 0.1 μg/ml was enough to stimulate the expression of vimentin. **b** A time-dependent increase of vimentin on the endothelial cells treated with 0.1 μg/ml VP1. **c** Immunofluorescence analysis showed the enhanced expression of vimentin on endothelial cells treated with VP1 for 24 h. **d** VP1 neutralization antibody competitively inhibited the roles of VP1 in the upregulation of vimentin. Ctrl-Ab control antibody, Anti-VP1 VP1 neutralization antibody. **e**, **f** Expression of EV-A71 receptors SCARB2, HSP-70, and Annexin II was similar in the different groups. PSGL-1 was decreased in the endothelia cells treated with VP1

Moreover, VP1 directly caused the elevation of vimentin on the brain endothelial cells (Fig. 4a–c), which was competitively inhibited by VP1 neutralization antibody (Fig. 4d). Meanwhile, roles of VP1 on the expression of EV-A71 receptors SCARB2, HSP-70, and Annexin II were negligible, and PSGL-1 was decreased on the VP1-treated brain endothelial cells (Fig. 4e, f). Collectively, VP1 may enhance the EV-A71 CNS infection via the upregulation of virus receptor vimentin on brain endothelial cells.

### TGF-/Smad3 and NF-κB were indispensable with vimentin expression

**Fig. 5:**
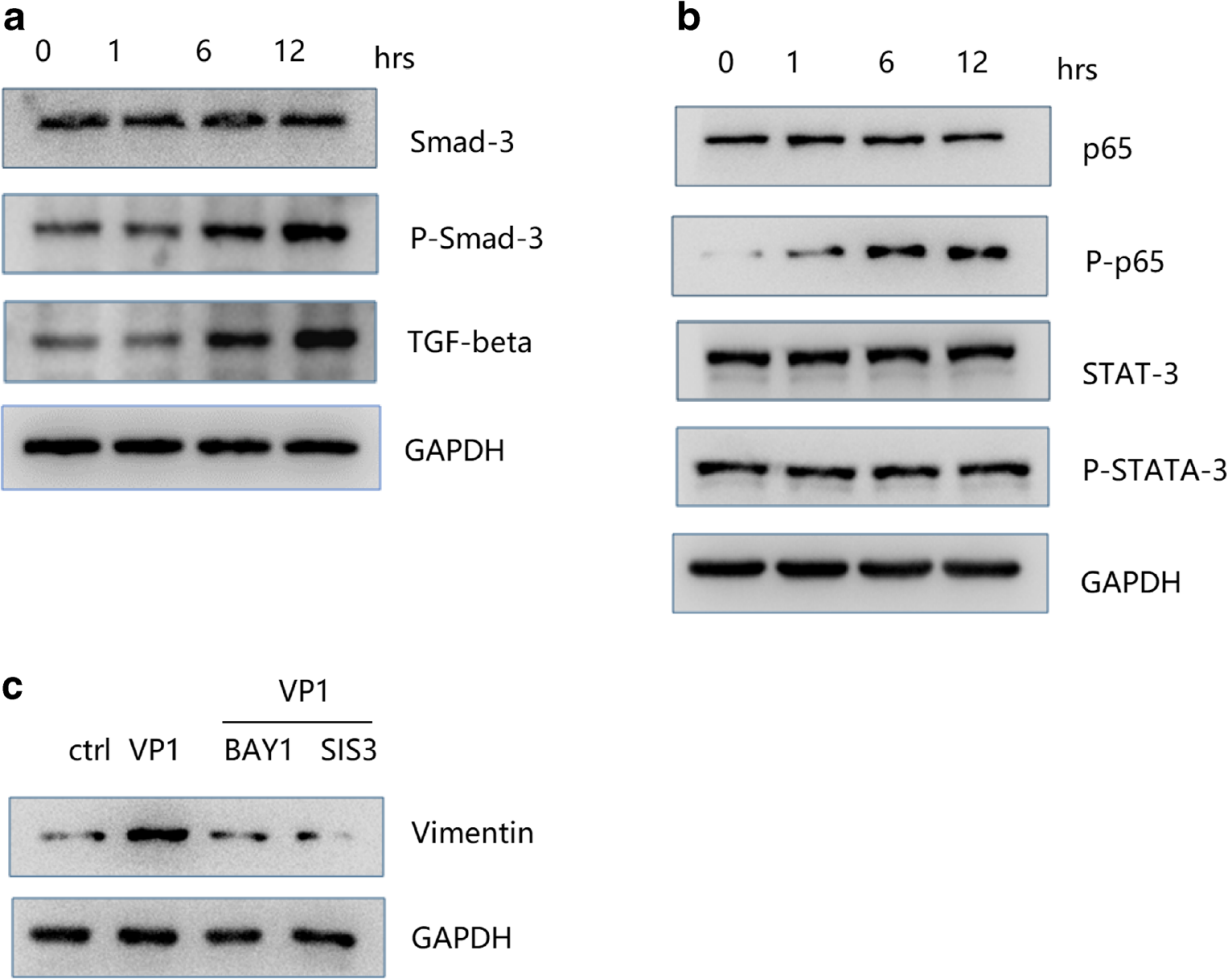
TGF-/Smad3 and NF-κB-dependent expression of vimentin on brain endothelial cells treated with VP1. **a** In the VP1-treated endothelial cells, TGF-β and Smad-3 was increased and activated. **b** NF-κB p65 was activated; and STAT3 was not changed. **c** NF-κB inhibitor BAY or Smad-3 inhibitor SIS3 suppressed the elevation of vimentin on the VP1-treated endothelial cells

Vimentin expression is regulated by diverse signal pathways, including TGF-β/Smad-3 (Wu et al. 2007), NF-κB (Kryszke and Vicart 1998), and STAT3 (Wu et al. 2004). To explore the mechanisms of vimentin upregulation, the brain endothelial cells were treated with VP1. In the VP1-treated endothelial cells, TGF-β and Smad-3 were increased, as well as the NF-κB p65 signal pathway; STAT3, however, was not changed (Fig. 5a, b). In addition, NF-κB inhibitor BAY or Smad-3 inhibitor SIS3 almost abolished the elevation of vimentin on VP1-treated endothelia cells (Fig. 5c). In sum, VP1 increased the expression of vimentin on brain endothelia cells, which was dependent on TGF-β/Smad-3 and NF-κB signal pathways.

### Vimentin over-expression was accompanied with BBB damage

**Fig. 6:**
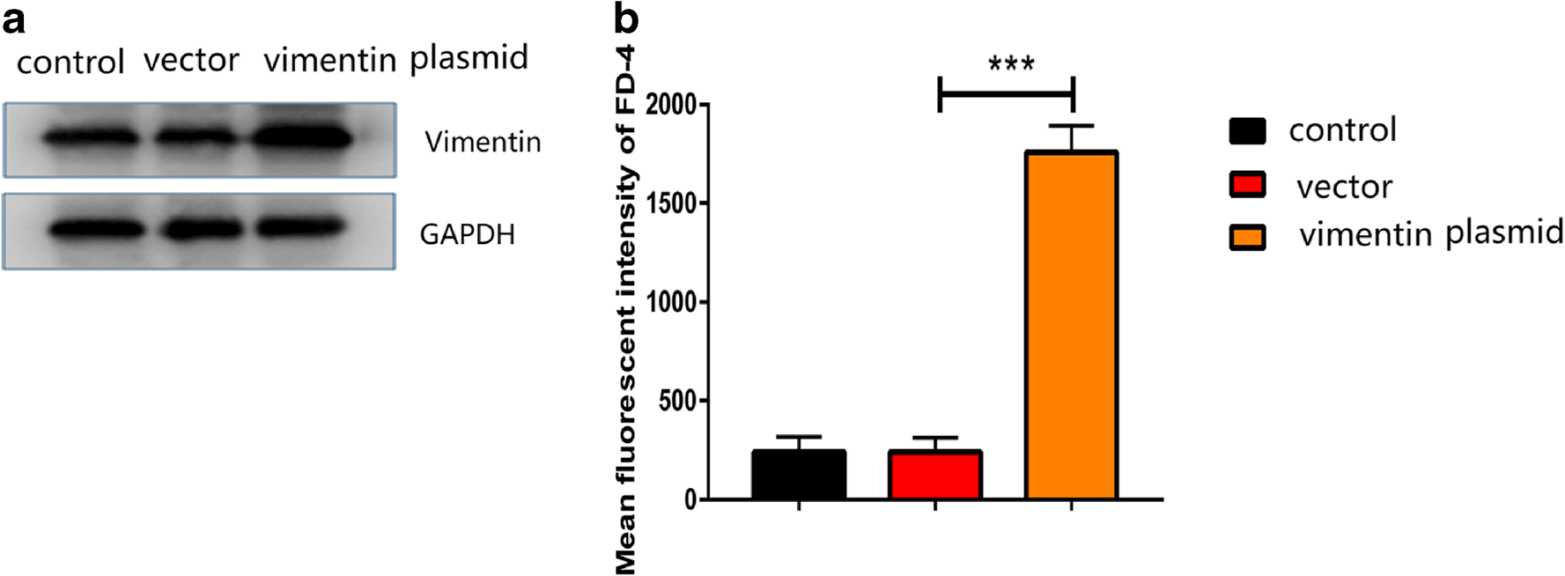
Vimentin over-expression damaged BBB integrity. **a** Vimentin expression plasmid promoted the expression of vimentin on brain endothelial cells. **b** Vimentin expression plasmid transfection increased the FD4 leakage through brain endothelial cell monolayer. ***p < 0.001

VP1 protein damaged the BBB integrity and increased the expression of vimentin on the brain endothelial cells. Therefore, the role of vimentin expression on the BBB integrity was explored. As shown in Fig. 6a, vimentin expression plasmid promoted the expression on brain endothelial cells. With the elevation of vimentin, more FD4 leaked through the BBB in vitro (Fig. 6b), suggesting that vimentin over-expression was accompanied with BBB damage at least in vitro.

## Discussion

Due to the fatal CNS syndrome in the infants, EV-A71 was considered as an important neurotropic virus (Rasti et al. 2019). Though inactivated virus vaccine eliciting safe and protective response against EV-A71 (Li et al. 2014) has been approved, the pathogenesis of EV-A71 CNS infection is still incompletely understood. In the present study, we demonstrated EV-A71 capsid protein VP1 directly damaged BBB integrity with the reduced claudin-5. Meanwhile, VP1 increased the virus receptor vimentin on brain endothelial cells through TGF-/Smad3 and NF-κB signal pathways. In addition, vimentin over-expression may lead to the increased leakage of BBB.

BBB impairment has been documented in patients infected with HIV-1 (Anesten et al. 2016), Japanese encephalitis virus (Li et al. 2015a), and other neurotropic viruses (Spindler and Hsu 2012). Similar with HIV-1 envelope protein gp120 compromised BBB integrity (Kanmogne et al. 2007), EV-A71 capsid protein VP1 damaged the intact BBB, which was demonstrated with elevated albumin and dextran in the brain parenchyma. Albumin (~ 65 kD), Evans blue, and fluorescence dextran (3~5 kD) were surrogate makers for the BBB impairment (Saunders et al. 2015). In the EV-A71 patients (Huang et al. 2010) or neonatal mice (Jin et al. 2018), vascular endothelial growth factor (VEGF) was significantly increased, which may decrease claudin-5 expression on brain endothelial cells (Argaw et al. 2009). In the line with compromised BBB, tight junction protein claudin-5 in the mice treated with VP1 was decreased. As a major tight junction protein in brain endothelial cells, claudin-5 was unique in the selective regulation of small molecules (< 800 D) across BBB (Nitta et al. 2003). Therefore, decreased expression of claudin-5 on brain endothelial cells may be not the sole reason for the VP1 induced leakage of albumin (~ 65 kD) and fluorescence dextran (3~5 kD) into brain parenchyma. We hypothesized that decreased claudin-5 and other factors may jointly contribute to the BBB impairment.

With the elevated leakage of BBB, EV-A71 viron may paracellularly invade the brain parenchyma through the abnormally loosened tight junctions. As one of EV-A71 virus receptors for VP1 (Du et al. 2014), vimentin was primarily expressed in endothelial cells and. Moreover, VP1 caused the vimentin rearrangement in astrocyte cells, facilitating virus infection in the CNS (Haolong et al. 2013). In the consideration that endothelial cells, vimentin is utilized by various viruses, including at least cowpea mosaic virus (Koudelka et al. 2009), dengue virus (Yang et al. 2016), and severe acute respiratory syndrome coronavirus (Yu et al. 2016), we speculated that VP1 may exploit endothelial cells and vimentin. In the accordance with the above speculation, VP1 activated TGF-β/Smad-3/NF-κB pathways, leading to the increased expression of vimentin in brain endothelial cells. The exact mechanisms of VP1 upregulating vimentin on brain endothelia cells, however, warranted further elucidation. Besides the virus receptor, vimentin also functioned as intracellular chaperone for EV-A71 protein 2C and promoted virus survival (Gladue et al. 2013). Paradoxically, inflammasome activation in the brain tissues was alleviated in the vimentin-deficient mice infected with EV-A71 (Xiao et al. 2018), suggesting that vimentin was necessary in the inflammation induction. No matter how vimentin was involved with EV-A71 pathogenesis, our results provided the possibility that EV-A71 may transcellularly transmigrate across BBB via the vimentin attachment on the brain endothelial cells.

BBB disruption was essential in the neurotropic viral infection (Al-Obaidi et al. 2018). As a protein with many divers aspects (Danielsson et al. 2018), vimentin played important roles in health and disease. In the cremaster muscle, vasculatures from vimentin-deficient mouse endothelial integrity were compromised (Nieminen et al. 2006), and in the pulmonary endothelia cells, vimentin redistribution and phosphorylation increased barrier permeability (Liu et al. 2014). We hypothesized that vimentin over-expression may lead to the junction proteins redistribution, therefore compromising the BBB integrity.

## Conclusion

This study revealed that the EV-A71 capsid protein VP1 increased blood–brain barrier permeability and virus receptor vimentin on the brain endothelial cells, which may benefit the virus entrance into brain parenchyma and cause fatal CNS diseases. We could not preclude the possibility that VP1 may also affect the retrograde axonal transport of EV-A71 by nerves.

## Data Availability

The datasets used and/or analyzed during the current study are available from the corresponding author on reasonable request.

## Abbreviations

*EV-A71*: enterovirus A71
*HFMD*: hand-foot-and-mouth diseases
*CNS*: central nervous system
*BBB*: blood–brain barrier
*HIV-1*: human immunodeficiency virus type 1
*SCARB2*: scavenger receptor class B member 2
*PSGL-1*: P-selectin glycoprotein ligand1
*HSP-70*: heat shock protein-70
*NS*: natural saline
*FITC*: fluorescein isothiocyanate
*DMSO*: dimethyl sulfoxide
*PVDF*: polyvinylidene difluoride
*DAPI*: 4′6-diamidino-2-phenylindole
*TEER*: trans-endothelial electrical resistance
*PBS*: phosphate-buffered saline
*SEM*: standard error of the mean
*VEGF*: vascular endothelial growth factor
